# Pleomorphic adenoma of the parapharyngeal space

**DOI:** 10.1016/j.radcr.2024.11.027

**Published:** 2024-12-19

**Authors:** Anas Douami, Oussama Marsafi, Aicha Merzem, Hasnaa Belgadir, Omar Amriss, Nadia Moussali, Naima Elbenna

**Affiliations:** Radiology Department -University Hospital of 20 Aout 1953 - Faculty of Medicine and Pharmacy of Casablanca, Hassan II University, Casablanca, Morocco

**Keywords:** Pleomorphic adenoma, Parapharyngeal space, CT scan, MRI

## Abstract

Pleomorphic adenoma of the parapharyngeal space is a rare benign tumor, representing less than 1% of tumors of the parapharyngeal space. Considering the volume of the tumor, the complexity of the space and the potential difficulties of excision, an imaging work-up including at least computed tomography (CT) and/or magnetic resonance imaging (MRI), and possibly analysis of the vascular axes (angio-MRI, arteriography) is essential. Treatment of these tumors is essentially surgical. We report a case of pleomorphic adenoma of the parapharyngeal space in a 38-year-old woman.

## Introduction

Tumors of the parapharyngeal space are rare, accounting for 0.5% of all head and neck tumors. They are benign in 70%-80% of cases, in different series. Pleomorphic adenomas or mixed tumors are more common in the parotid gland. It is defined as a heterogeneous benign tumor of the salivary glands. It is rare in the parapharyngeal space. We report the case of a ‘pleomorphic adenoma of the left parapharyngeal space, diagnosed on the basis of pathognomonic features on cross-sectional imaging (CT-MRI) and biopsy.

## Case report

We report a case of a 38-year-old woman who was seen in consultation for a left jugal tumefaction evolving for 3 years without any alteration of the general state or notion of fever. Physical examination revealed a left jugal tumefaction with bulging of the palate associated with anterior and medial displacement of the homolateral tonsil palate and anterior pillar.

There were no other associated signs, notably no neurological deficit, otological sign or cervical adenopathy.

Nasofibrosocopy revealed regular bulging of the left posterolateral wall of the cavum.

**Fig. 1:**
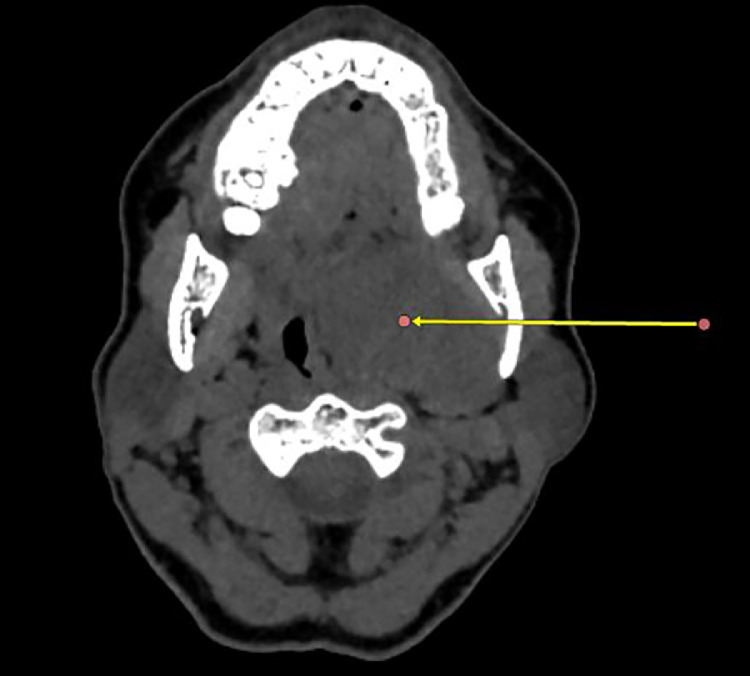
Axial CT scan, wide-window view, showing a well-bounded tissue mass in the left parapharyngeal space, separated from the deep lobe of the parotid gland by a fatty border .

A facial CT scan without and with injection revealed a well-bounded tissue mass in the left parapharyngeal space, separated from the deep lobe of the parotid gland by a fatty border. The mass enhanced heterogeneously after contrast injection. There was no bone lysis (Fig. 1).

**Fig. 2:**
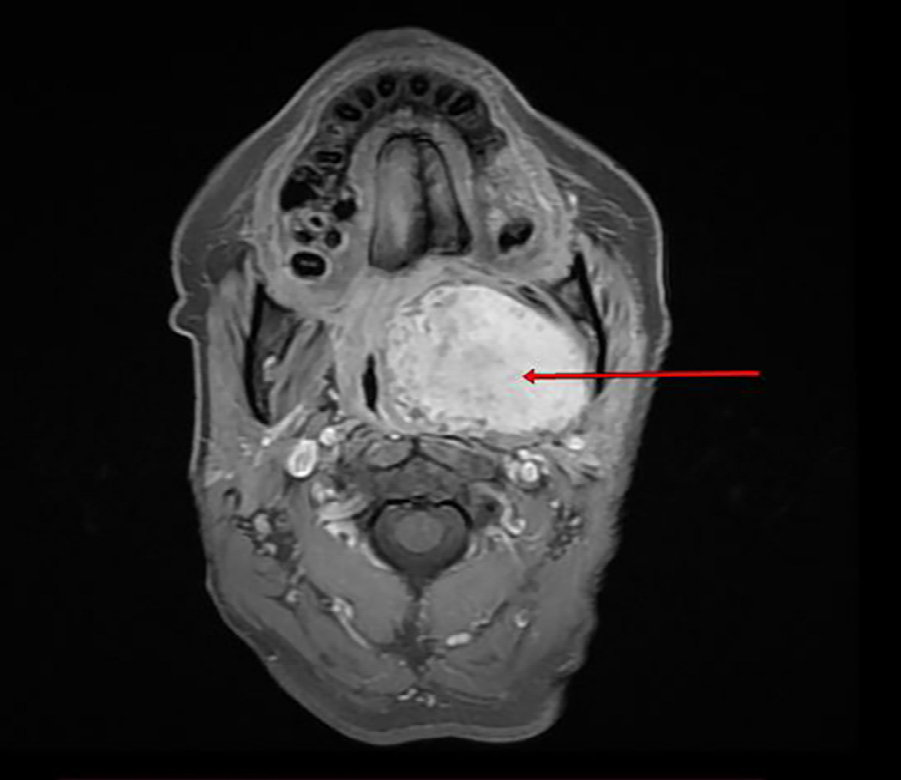
Axial T1-weighted image FATSAT with Gadolinium injection showing a hyporintense mass in the left parapharyngeal space .

**Fig. 3:**
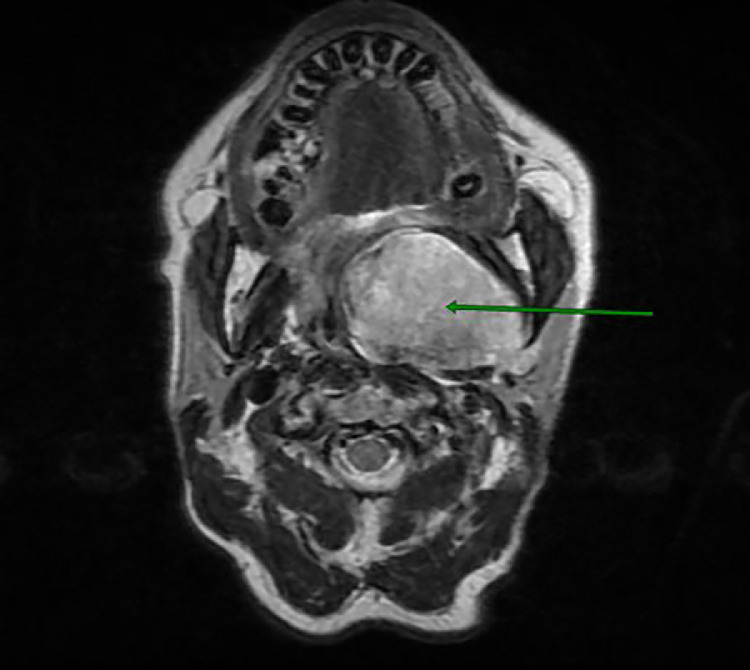
Axial T2-weighted image showing a hyperintense lesion in the left parapharyngeal space .

**Fig. 4:**
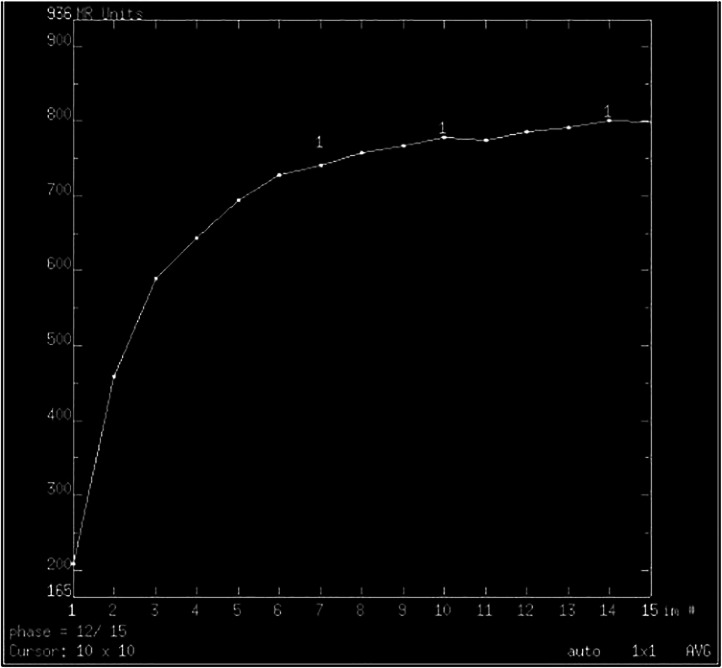
An ascending tray to the type A perfusion sequence.

**Fig. 5:**
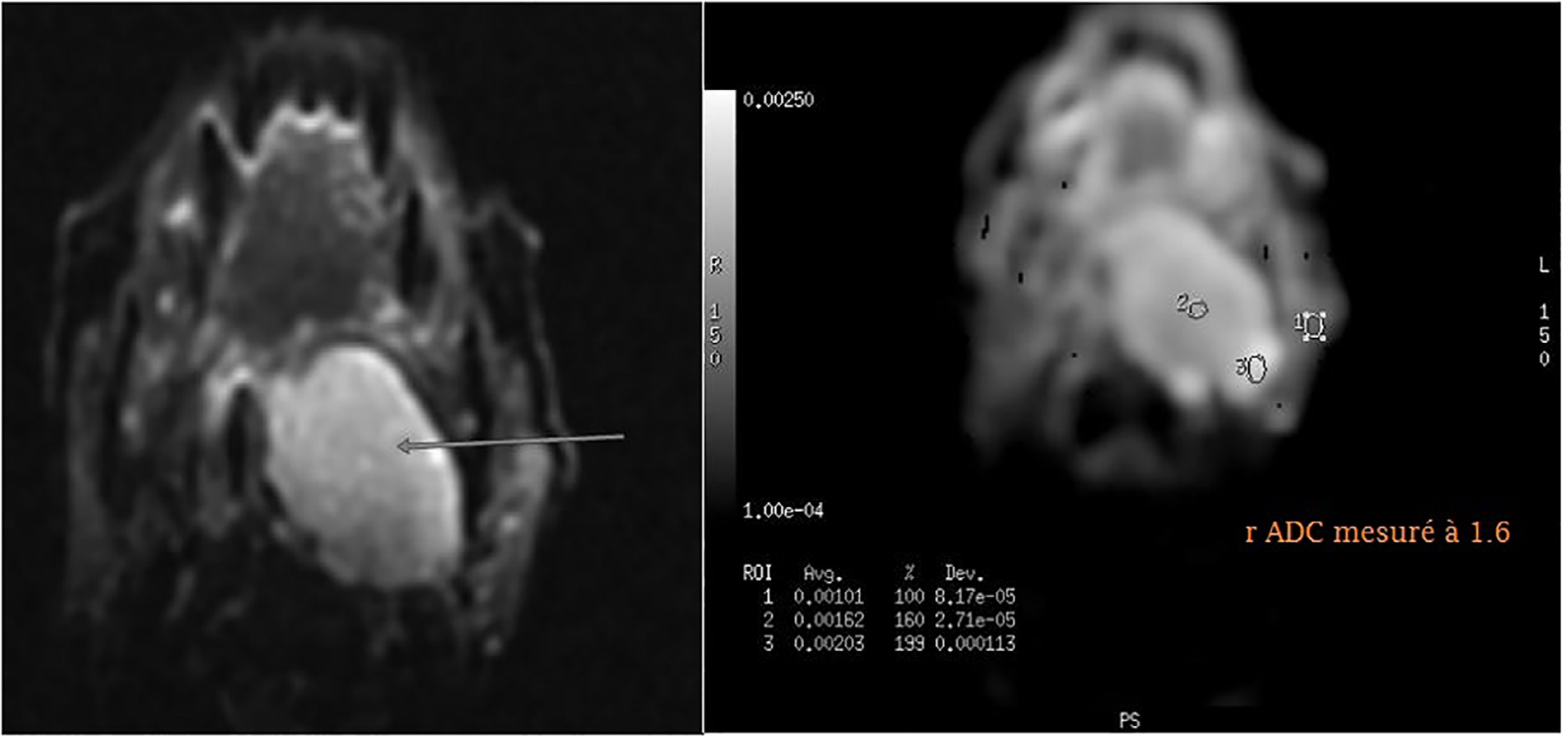
Axial image diffusion with hypersignal and r ADC > 1.3.

MRI revealed a well-limited, heterogeneous mass with T1 iso-signal and T2 hyper-signal and diffusion with r ADC > 1.3, intensely and heterogeneously enhancing after injection of Gadolinium with an ascending plateau on the perfusion sequence. (Fig. 2, Fig. 3, Fig. 4, Fig. 5).

The preliminary diagnosis, based on CT and MRI findings, was in favor of a pleomorphic adenoma of the parapharyngeal space.

The diagnosis was confirmed by biopsy in favor of a pleomorphic adenoma of the parapharyngeal space.

Surgical excision was performed via anterior cervical approach; the patient had an excellent response and remains asymptomatic and in excellent clinical condition.

## Discussion

Tumors of the parapharyngeal space are characterized by a great histological diversity, explained by the different structures contained in this space [1]. These tumors are dominated by tumors of the salivary glands. The pleomorphic adenoma is a heterogeneous benign tumor and the most frequent of the salivary gland tumors [2,3]. Long referred to as a mixed tumor due to its dual epithelial and mesenchymal component. Its extra-parotid localizations are rare [[4], [5], [6]].

Clinical expression is delayed due to its deep-seated location. Other signs correlate with the tumor's mass effect on adjacent structures, such as dysphagia, pharyngeal discomfort, rhinolalia and snoring. Sleep apnea syndrome has also been described. The physical examination is poor. It usually reveals a non-beating intraoral mass covered by a smooth mucosa pushing the tonsil medially [[7], [8], [9]].

A CT scan of the face, with and without injection of contrast product, with reconstruction in the 3 planes of space, is the examination of first line. It objectifies the mass, examines its relationship with the skull base, studies its vascularization and pinpoints its pre- or retrostylial position [10].

MRI is an important examination in the workup of parapharyngeal tumors [[11], [12], [13], [14]]. The possibility of obtaining images in frontal, axial and sagittal sections is a significant contribution to good spatial assessment. T1-weighted images allow good visualization of normal anatomy and peritumoral fat margins, while T2-weighted images show tumor margins and the tumor-muscle interface. On T2-weighted slices, it shows a fatty border separating the tumor from the deep lobe of the parotid gland in the case of a primary pleomorphic adenoma of the accessory salivary glands. Its sensitivity in the topographical diagnosis of salivary tumors in the parapharyngeal space varies between 95% and 100%.

Two functional sequences are available: an axial diffusion sequence up to b1000 with ADC mapping, and a dynamic T1-weighted perfusion sequence performed after injection of Gadolinium, enabling tumor enhancement to be followed over time [[15], [16], [17]].

Signs in favor of a benign lesion are: well-limited contours, the presence of intra-tumoral hypersignals on spontaneous T1 weighting, a very intense pseudo-liquid T2 franchomogenous intra-tumoral hypersignal, the absence of associated adenopathy, and regularity with clear limits. Conversely, signs in favor of an aggressive tumor are irregular contours, infiltration of perilesional fat, frank T2 tumoral hyposignal and the presence of satellite adenopathies. Some lesions, however, remain indeterminate on morphological criteria alone, such as cystic adenoid carcinoma, which may be T2 hypersignal and therefore difficult to differentiate from a pleomorphic adenoma, or may be the site of hemorrhagic necrosis causing T1 hypersignals, mimicking a Warthin tumor. In such cases, functional sequences are essential, as they will provide additional arguments in favor of malignancy [18].

Diffusion sequences with ADC mapping provide information on the degree of cellularity of a lesion. These sequences are analyzed using dedicated software, by measuring the diffusion coefficient on a non-necrotic, non-hematic tissue zone. A diffusion hypersignal with ADC restriction is indicative of a hypercellular lesion. A particular feature of parotid tumor analysis is the measurement of rADC: the ratio between ADC within the tumor and within the healthy homo or contralateral parotid, thus defining threshold values for distinguishing between tumors . An rADC of less than 1 indicates a malignant tumor. A very low rADC, around 0.5, is more likely to be lymphoma [19].

An rADC greater than 1.3 is in favor of a pleomorphic adenoma, and therefore a benign tumor.

An rADC between 1 and 1.3 favors a hypercellular pleomorphic adenoma or a tumor of intermediate malignancy.

Only Warthin's tumor (benign) has an rADC of less than 1. However, perfusion curves and morphological imaging can help to correct the diagnosis.

Macroscopically, the tumor is nodular, well circumscribed or even encapsulated by a conjunctive gangue, and is usually whitish-gray in color, sometimes translucent on section. Consistency varies from firm to soft and gelatiniform. Its pleomorphic character refers to its architectural richness, in contrast to the monomorphism of its epithelial and myoepithelial cells. These cells are usually regular and cytologically “reassuring”. An important element of the diagnosis is the observation of a particular stroma which, very characteristically, takes on a myxoid appearance, sometimes with cartilaginous or bony differentiation [20,21].

Treatment is essentially surgical: several approaches have been described, but the best one must allow good exposure in order to identify and protect the vascular-nervous structures, while ensuring complete removal of the tumor [[22], [23]].5% of pleomorphic adenomas have been reported in the literature to have a postoperative recurrence rate. This may be due to fragmentation of the tumor during excision [23].

The prognosis is generally good, but there is a high risk of recurrence after surgery and of carcinomatous degeneration, requiring early surgical management and regular surveillance [24].

## Conclusion

Pleomorphic adenoma is a heterogeneous benign tumor of the salivary glands. Its extra-parotid localization is rare. The management of pleomorphic adenomas of the parapharyngeal space requires a complete radiological work-up (CT and MRI), which allows us to diagnose the location of the tumor, guide the choice of approach and orient the histological diagnosis. Treatment is surgical. The distinction between tumors of the accessory salivary glands and tumors of the deep lobe of the parotid gland is necessary to guide the choice of approach.

## Author contributions

All the authors contributed to the conduct of this research work. The authors have read and approved the final version of the manuscript.

## Patient consent

I confirm that I have obtained the patient's consent for the publication of this article.
